# RNA-seq reveals the critical role of OtpR in regulating *Brucella melitensis* metabolism and virulence under acidic stress

**DOI:** 10.1038/srep10864

**Published:** 2015-08-05

**Authors:** Wenxiao Liu, Hao Dong, Jing Li, Qixing Ou, Yujin Lv, Xiaolei Wang, Zuoshuang Xiang, Yongqun He, Qingmin Wu

**Affiliations:** 1 Key Laboratory of Animal Epidemiology and Zoonosis of Ministry of Agriculture, College of Veterinary Medicine, China Agricultural University, Beijing 100193, People’s Republic of China; 2 Unit for Laboratory Animal Medicine and Department of Microbiology and Immunology, The University of Michigan Medical School, Ann Arbor, MI 48109; 3 Center for Computational Medicine and Bioinformatics, The University of Michigan Medical School, Ann Arbor, MI 48109; 4 Beijing Senkang Biotech Development Co., LTD, Beijing 101400, People’s Republic of China; 5 Department of Zoonosis Control, China Animal Disease Control Center, Beijing, People’s Republic of China; 6 Department of Veterinary Medicine, Zhengzhou College of Animal Husbandry Engineering, Zhengzhou 450046, People’s Republic of China

## Abstract

The response regulator OtpR is critical for the growth, morphology and virulence of *Brucella melitensis*. Compared to its wild type strain 16 M, *B. melitensis* 16 MΔ*otpR* mutant has decreased tolerance to acid stress. To analyze the genes regulated by OtpR under acid stress, we performed RNA-seq whole transcriptome analysis of 16 MΔ*otpR* and 16 M. In total, 501 differentially expressed genes were identified, including 390 down-regulated and 111 up-regulated genes. Among these genes, 209 were associated with bacterial metabolism, including 54 genes involving carbohydrate metabolism, 13 genes associated with nitrogen metabolism, and seven genes associated with iron metabolism. The 16 MΔ*otpR* also decreased capacity to utilize different carbon sources and to tolerate iron limitation in culture experiments. Notably, OtpR regulated many *Brucella* virulence factors essential for *B. melitensis* intracellular survival. For instance, the *virB* operon encoding type IV secretion system was significantly down-regulated, and 36 known transcriptional regulators (*e.g., vjbR* and *blxR*) were differentially expressed in 16 MΔ*otpR.* Selected RNA-seq results were experimentally confirmed by RT-PCR and RT-qPCR. Overall, these results deciphered differential phenomena associated with virulence, environmental stresses and cell morphology in 16 MΔ*otpR* and 16 M, which provided important information for understanding the detailed OtpR-regulated interaction networks and *Brucella* pathogenesis.

*Brucella spp.* is a group of facultative intracellular bacteria^1^. The virulence of *Brucella* depends on their ability to survive in professional and non-professional phagocytes. The intracellular niche of phagocytes contains various harsh environments like nutrition deprivation, low-pH conditions, and low-oxygen tension^2^,^3^,^4^. *Brucella* use several transcription regulators, including OtpR^5^, NtrYX^6^, and Rsh^7^, to activate a series of interacting signal networks in response to these environmental stresses. Among these transcription regulators, our studies first found that *Brucella* OtpR (BMEI0066) regulates stress responses, cell growth and cell morphology of *Brucella*^5^,^8^. *Brucella* OtpR (BMEI0066) is a cytoplasmic protein that shows significant similarity to the OmpR subfamily with two conserved domains: a signal receiver domain with a phosphoacceptor site and an effector domain with DNA-binding activity^9^. Our previous experiments demonstrated that 16 MΔ*otpR*, an *otpR* mutant of the virulent *B. melitensis* strain 16 M, was avirulent in mice and had a reduced capacity to invade phagocytic cells^8^. Compared with its parental strains, 16 MΔ*otpR* displayed an unusual, irregular deformation of the cell envelope^8^. These findings suggested that OtpR could regulate the cell morphology and cell growth. The expression of some cell division–associated proteins, including FtsQ, is reduced in 16 MΔ*otpR* as compared with the parental strain^8^. However, the breadth of genes and the gene networks that are regulated by OtpR are still unclear.

One major mechanism of *Brucella* pathogenesis is the capacity of virulent *Brucella* surviving in an acidic environment inside macrophages^10^,^11^. Porte *et al.* found that early acidification of phagosomes containing *B. suis* is essential for intracellular survival inside macrophages^4^. The pH in the phagosomes containing live *B. suis* decreased to values of pH 4.0 ± 0.5. At 1 h postinfection, the phagosome was already acidic and remained acidic for at least 5 h. An early neutralization of vacuolar pH in fact inhibits the survival of *B. suis* inside macrophages^4^. *Brucella* is able to resist well to an acidic condition of pH 3.2 for several hours inside macrophages^12^. Similarly, virulent *B. abortus* also survives well inside an acidic intracellular condition^13^. Boschiroli *et al.* found that transcription of *Brucella virB* operon, which encodes for the Type IV secretion system (T4SS), was induced specifically within macrophages, and the phagosome acidification is a key intracellular signal inducing *virB* expression^14^. *B. melitensis* was found to induce a specific set of proteins (*e.g.*, DnaK) in response to acidic pH^15^. Similar findings have also been reported in other bacteria. For example, *Salmonella typhimurium* activates virulence gene transcription within acidified macrophages^16^. These studies indicate that low pH acts as an intracellular signal on the regulation genes involved in survival and multiplication within phagocytic cells^4^,^16^. However, the detailed mechanism of the regulatory gene network of *Brucella* under acidic environment is still unclear.

Our previous study has observed that acid stress induces an approximately 1.5-fold increase in OtpR expression, suggesting that OtpR is activated under acid stress. 16 MΔ*otpR* has a significantly reduced capacity in response to acid stress^5^. As described above, OtpR is also critical to regulate cell morphology and cell growth and several proteins (e.g., FtsQ). In addition, many other proteins such as T4SS proteins^14^, DnaK^15^, VjbR^17^, and Hfq^18^, are also regulated at acidic conditions. Therefore, to further define the OtpR-regulated *Brucella* pathogenesis mechanism, we hypothesized that OtpR plays a major role as an important transcription regulator in regulating *Brucella* genes critical for intracellular survival under an acidic condition. Our previous study has observed that acid stress induces an approximately 1.5-fold increase in OtpR expression, suggesting that OtpR is activated under acid stress. 16 MΔ*otpR* has a significantly reduced capacity in response to acid stress^5^. As described above, we also found that OtpR is critical to regulate cell morphology and cell growth and several proteins (*e.g.*, FtsQ). In addition, many other proteins (*e.g.*, T4SS proteins, DnaK) were also regulated at acidic conditions (see the description above). Therefore, to further define the OtpR-regulated *Brucella* pathogenesis mechanism, we hypothesized that OtpR play a major role as an important transcription regulator in regulating *Brucella* genes critical for intracellular survival under an acidic condition. To address this hypothesis, it would be ideal to use a high throughput technology to detect and compare the gene expression profiles of 16 MΔ*otpR* and its parental strain 16 M under an acid stress. Through high efficient sequencing of complementary DNAs (cDNAs) that are reverse transcribed from RNAs, the RNA-seq technology has many advantages compared to the microarray technology in whole-genome gene expression analysis^19^,^20^. RNA-seq does not require probe sequences and also has a greater dynamic range for measuring very low or very high gene expression levels^21^. The power of RNA-seq has been demonstrated in the transcriptomics studies for *Brucella*^22^,^23^,^24^ and many other bacteria^19^,^20^,^21^. Therefore, we used RNA-seq in current study. The results provided fundamental gene-level evidence and detailed gene expression profiles regulated by OtpR in *Brucella.*

## Materials and Methods

### Bacteria strains

Bacterial strains used in the present study were *B. melitensis* 16 M, *B. melitensis* 16 MΔ*otpR,* 16McΔ*otpR.* Strain 16 M is a commonly used, virulent, wild type *B. melitensis* strain. 16 MΔ*otpR* is the *otpR* mutant of 16 M that has an avirulent phenotype. 16McΔ*otpR* is the virulent complementation strain of 16 MΔ*otpR.* Both 16 MΔ*otpR* and 16McΔ*otpR* were generated in our laboratory and previously reported^8^.

### Bacterial growth and RNA preparation

*Brucella* 16 M and 16 MΔ*otpR* were grown with 100 mL of Tryptic Soy Broth (TSB; BD; final pH = 7.3) in a 500-mL water-bath shaker (180 rpm) at 37 °C until early-log phase (OD_600_  ≅ 0.6 − 0.7). The acid treatment experiment followed the same protocol as previously reported^5^. Under this protocol, the cells were treated with the same TSB medium but with an acidic condition (pH 3.4 − 4.4)^5^. After the treatment, the cell cultures were collected and centrifuged. After the centrifugation, the supernatants were removed, and the RNA protect Bacteria Reagent (Qiagen, Hilden, Germany) was added to the pellets to prevent RNA degradation.

The *B. melitensis* RNAs for Solexa/Illumina sequence were isolated and purified with RNeasy Mini System (Qiagen, Hilden, Germany). RNA was eluted from the column using RNase-free water. Total RNA was incubated with DNase (Ambion, Foster City, CA) and then purified using two phenol-chloroform extractions and one chloroform extraction. RNA was resuspended in RNAase free TE buffer (10 mM Tris, 1 mM EDTA; pH 8.0; Ambion). The purity and integrity of RNA was assessed using the 2100 Bioanalzyer (Agilent Technologies, Palo Alto, CA, USA). *B. melitensis* mRNA was enriched by removal of 16 S and 23 S rRNA from two 5 μg aliquots of total RNA using a MicrobExpress Bacterial mRNA purification Kit (Ambion). As ≤ 5 μg total RNA was treated per reaction, a separate enrichment reaction was performed for each RNA sample to enrich the RNA volume for the subsequent experiments. The mRNA sample was assessed with the 2100 Bioanalyzer to confirm the reduction of 16 S and 23 S rRNAs prior to the preparation of cDNA fragment libraries.

### cDNA library preparation and sequencing using the Illumina Genome Analyzer

The RNA was subjected to Solexa/Illumina sequencing at Beijing Genomics Institute. The cDNA library was constructed as previously^25^. Briefly, each mRNA sample was fragmented into short sequences with divalent cations and heat^25^. Using these short fragments as templates, the first-strand cDNA was synthesized with random hexamer primers and reverse transcriptase (Invitrogen, Carlsbad, CA). The second-strand DNA was synthesized using RNase H (Invitrogen) and DNA polymerase I (New England Biolabs, Beverly, MA, USA), respectively. The amplified fragments were purified with QiaQuick PCR Purification kit (Qiagen, Hilden, Germany) and resolved with EB buffer for the end preparation and poly (A) addition. Individual paired-end libraries for each sample were constructed and loaded onto independent flow cells. Sequencing was carried out by running 35 cycles on the Illumina HiSeq 2000 platform.

Raw 90-bp sequence data were generated using the Illumina Genome Analyzer II system. All sequences were examined for possible sequencing errors. The raw sequence data was filtered by removing reads that contained adaptor sequences, consisted of >5% ambiguous residues (Ns), or had the majority base quality of <5. The raw data have been submitted to the National Center for Biotechonology Information-Gene Expression Omnibus (NCBI GEO) database http://www.ncbi.nlm.nih.gov/geo/). The Accession ID is GSE48165.

### RNA-seq alignment and identification of transcribed and annotated CDS

To increase the quality of the reads, the raw reads with the length of 90-bp each were trimmed to 75 bp after quality evaluation using FastQC (http://www.bioinformatics.babraham.ac.uk/projects/fastqc/). The trimmed reads were aligned with the *B. melitensis* 16 M genome (NC_003317 and NC_003318) and annotated gene sets obtained from NCBI (ftp://ftp.ncbi.nlm.nih.gov/genomes/) using the Short Oligonucleotide Analysis Package (SOAP)^26^. cDNAs with matches to the reference genome of >80% were retained for further analysis. Those sequencing reads that matched annotated genes in the *B. melitensis* 16 M genome reflect the genes transcribed under the given experimental conditions. Gene expression was quantified as Reads Per Kilobase of coding sequence per Million reads RPKM algorithm^27^. A gene was considered to be differentially expressed if the difference in RPKM values between the two samples (16 M and 16 MΔ*otpR*) was ≥2.0-fold (i.e., log_2_ ratio >1.0) and the p-value was <0.05^27^.

### COG category and Pathway analysis of the otpR-dependent genes

The next generation sequencing method resulted in the identification of the transcription levels of genes in 16 MΔ*otpR* and 16 M under acid stress. All possible *otpR-*dependent genes were identified using statistical methods as performed in a previous study with modifications^28^. COG annotations for the chosen genes were obtained from NCBI COG database (http://www.ncbi.nlm.nih.gov/COG/). The program OntoCOG was used for the COG enrichment test as previously described^29^.

### Reverse Transcriptase-polymerase Chain Reaction (RT-PCR) and Quantitative Real-time PCR (RT-qPCR) analyses

To confirm the RNA-Seq results, 28 up- or down-regulated genes from the RNA-Seq analysis were selected, and RT-PCR and RT-qPCR were carried out to confirm the gene expression changes on these 28 genes. PCR primers were designed using Primer 5.0 software (Primer-E Ltd., Plymouth, United Kingdom) and are listed in Supplemental Data 1. The same experimental protocols were used to culture both wild type 16 M and 16 MΔ*otpR* and extract RNA samples. The immunofluorescence analysis was performed with SYBR Green Master Mix (Applied Biosystems, Foster City, CA) using the 7500 Real Time PCR System (Applied Biosystems) as previously described^8^. Relative gene expression was calculated by the 2^–∆∆Ct^ method^8^. All reactions were carried out in triplicates.

### Stress challenge assays

To monitor extracellular growth under limited nutrition, the minimal medium (0.5% lactic acid, 3% glycerol, 0.75% NaCl, 1% K_2_HO_4_, 0.01% Na_2_S_2_O_3_·5H_2_O, 10 μg/ml Mg^++^, 0.1 μg/ml Fe^++^, 0.1 μg/ml Mn^++^, 0.21 μg/ml thiamine·HCl, 0.2 μg/ml nicotinic acid, 0.04 μg/ml calcium pantothenate, 0.001 μg/ml biotin, 5 mg/ml glutamate; pH 6.8–7.0 with NaOH) was inoculated with 10^6^ colony forming units (CFU)/ml of the 16 MΔ*otpR*, 16 M, or 16McΔ*otpR* strains. The cultures were incubated at 37 °C, and growth was monitored based on the OD_600_.

To assess the survival of 16 MΔ*otpR* in iron-limited medium, the bacteria were grown in TSB medium with a range of concentrations of the Fe^2+^chelator 2,2`-dipyridyl (DIP; Sigma-Aldrich, Shanghai, China) with an initial density of 3.0 × 10^7^ CFU/ml^30^. CFUs were determined at 24, 48, and 72 h after inoculation. All assays were performed in triplicates.

### Statistical analysis

The differences between the means of gene expressions for the experimental and control groups were analyzed by the Student’s unpaired t-test (equal sample sizes, equal variance) using SPSS 18.0. For the RNA-seq study, the P-values with the FDR (False Discovery Rate) multi-test adjustment were used to determine the differential expressed genes in the experimental groups compared to the control groups. The FDR P-value ≤0.001 and the absolute value of log_2_Ratio ≥1 (i.e., 2-fold change) were used as the thresholds to identify the genes showing statistically significant gene expression changes. For the RT-PCR study, P-value <0.05 was considered as statistically significant. The Student’s unpaired t-test was also used to analyze the bacterial survival rates under a stress condition.

## Results

### OtpR differentially regulated 501 genes in *B. melitensis*

**Figure 1:**
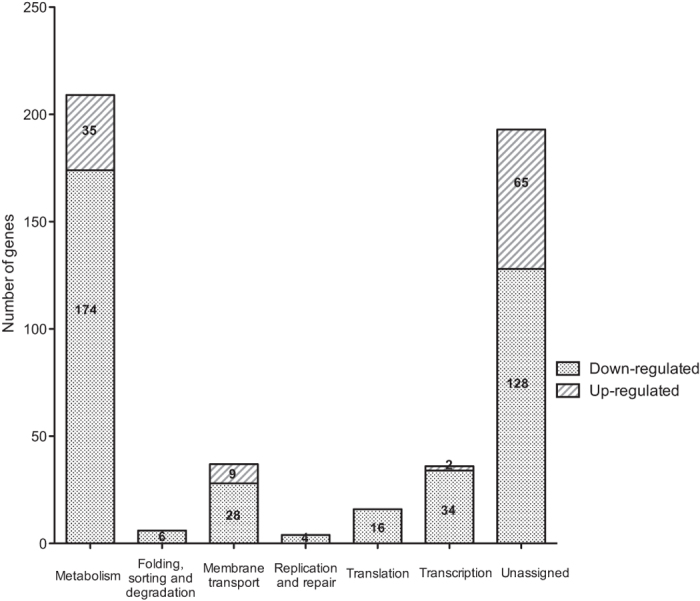
Functional categories of the differentially expressed genes in the *otpR* mutant as compared with the wild-type strain. Only genes that were up- or down-regulated by ≥2.0-fold are shown.

To detect all the possible genes regulated by OtpR during acid stress, the Next-Generation Sequencing (NGS) technology was used to sequence the whole transcriptomic profiles of 16 MΔ*otpR* and its wild-type strain 16 M. The raw sequence output of the two strain transcriptomes included 150 million reads in total. Approximately 50% reads were perfectly matched to the reference genome *B. melitensis* 16 M. Based on the genomic alignment, our analysis determined the expression of 3,163 genes in each strain. In total, 501 genes in *B. melitensis* were identified to be differentially expressed in OtpR (Fig. 1 and Supplemental Data 2). Among these genes, 390 genes were down-regulated and 111 genes were up-regulated in 16 MΔ*otpR* compared to the 16 M control. Most of these differentially expressed genes were associated with carbohydrate metabolism (10.78%), energy metabolism (7.39%), amino acid metabolism (6.19%), nucleotide metabolism (4.59%), lipid metabolism (1.80%), membrane transport (7.39%) and transcription (7.19%) (Fig. 1).

It is noted that the traditional experiments typically determine relevant expression levels of the genes using internal housekeeping gene control (e.g., β-actin) to normalize the results^31^,^32^,^33^. However, the next-generation sequencing (NGS), including RNA-seq, counts the absolute numbers of sequence reads mapped to the genomes^34^,^35^. After the counting, the gene expression quantification was measured using the Reads Per Kilobase of coding sequence per Million reads (RPKM) algorithm^27^. With the RPKM algorithm, there is no need to have an internal control as typically seen in many traditional RT-PCR or microarray experiments. By comparing the expression values between the two samples (16 MΔ*otpR* vs 16 M control), we were able to identify which genes were significantly regulated by OtpR under the same experimental condition.

More specific analysis results of these up- and down-regulated genes are described below.

### OtpR regulates *Brucella* cell division and cell envelope generation

Our sequencing analysis found OtpR regulates many genes directly involved in the cell division cycle. For example, three filamentous temperature sensitive genes *ftsK* (BMEII0742), *ftsQ* (BMEI0582), and *ftsZ* (BMEI0585) were down-regulated in the *otpR* mutant 16 MΔ*otpR*. These genes encode for three cell division proteins FtsK, FtsQ, and FtsZ^36^. FtsK acts as a bifunctional protein: its C-terminal domain facilitates segregation of chromosome dimers and its N-terminal may acts in the developing septum^36^. FtsQ (BMEI0582) is a highly conserved protein of the bacterial divisome, which is critical in linking the upstream and downstream cell division proteins to form the divisome^37^. The GTP-binding protein FtsZ is the key factor in the initiation of cell division by the formation of a ring-shaped structure^38^. In addition, our study also found that OtpR up-regulated intracellular septation protein BMEI0130.

Fatty acids participate in a number of cellular processes, most importantly in generating the cell envelope. Five genes for fatty-acid biosynthesis (BMEI1180; BMEI1473; *fabG,* BMEII0514; BMEI1521; BMEI1522; *fadD,* BMEI1632; *cfa,* BMEI1484) were down-regulated in the 16 MΔ*otpR*, suggesting that OtpR up-regulates these five fatty-acid biosynthesis genes.

In addition to fatty-acid biosynthesis genes, OtpR regulates many other genes directly involving cell envelope protein generation, assembly, transport, and structure. In Gram-negative bacteria, lipoproteins are one of the most abundant proteins anchored to the outer membrane through the lipids, which regulates the bacteria-host interaction and intracellular survival^39^. Compared to strain 16 M, the lipoprotein *oprf* (BMEII0036) was 2.25-fold down-regulated in strain 16 MΔ*otpR.* The down-regulated lipoprotein might lead to the modification of the cell surface proteins^40^. Two chaperone proteins GroES and GroEL were detected to be down-regulated in 16 MΔ*otpR.* These chaperones mediated the protein folding and could stimulate an immune response of T cells^41^. The GroESL homologues belong to a family of selective stress proteins during the intracellular growth, which could be induced by many stress stimuli including acid shock, heat shock, or oxidative injury^42^,^43^. Other genes participating in cell envelope protein generation or transport, including an ABC transporter substrate binding protein (BMEI1954), *apbE* (BMEII1010), and bactoprenol glucosyl transferase (BMEII1101), were also down-regulated in 16 MΔ*otpR* compared to its wild type control.

In 16 MΔ*otpR,* eleven genes associated with ribosomal proteins were down-regulated. Ribosomal proteins are critical for protein production, cell replication, and bacterial growth.

### OtpR regulates carbon, nitrogen, and energy metabolism in *B. melitensis*

**Figure 2:**
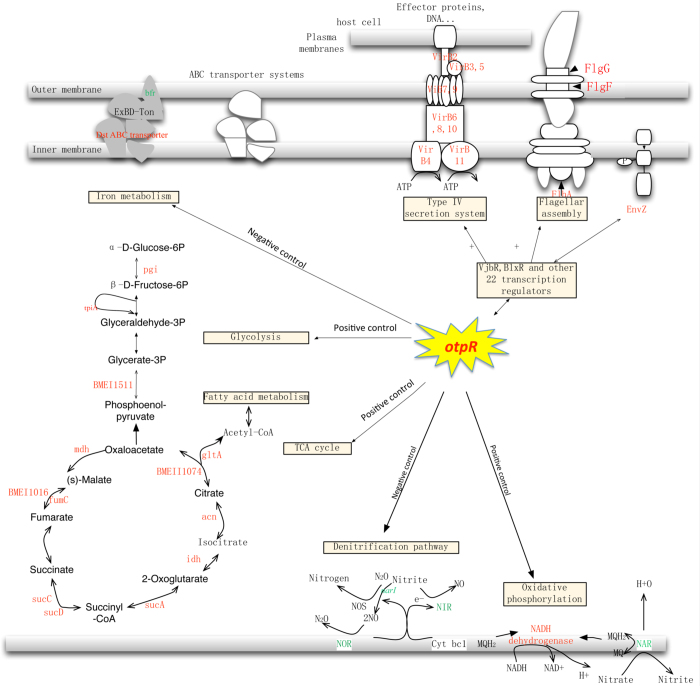
Basic metabolism regulated by *Brucella* OtpR identified in transcriptome analysis. Up-regulated genes in 16 MΔ*otpR* are shown in green and down-regulated genes in 16 MΔ*otpR* are shown in red.

The transcriptome analysis indicated that many genes associated with carbon and energy metabolism were significantly down-regulated in the *otpR* mutant under an acid stress. Most interestingly, these included twelve genes involved in the tricarboxylic acid (TCA) cycle (*mdh,* BMEI0137; *sucD,* BMEI0138; BMEI0139; *sucA,* BMEI0140; BMEI0791; *gltA,* BMEI0836; BMEI0855; BMEI0856; class I fumarate hydratase, BMEI1016; *acn,* BMEI1855; *fumC,* BMEII1051; and citrate lyase beta chain, BMEII1074; Fig. 2). The TCA cycle is critical for carbon metabolism and energy generation. The pyruvate metabolism supplies energy to living cells through the TCA cycle when oxygen is present (aerobic respiration), and alternatively through fermentation when oxygen is lacking^44^. Several genes relating the pyruvate metabolism were down-regulated in the *otpR* mutant, including *mdh* (BMEI0137), FAD-linked oxidase (BMEI0599), *pdhB* (BMEI0855), and *aceF* (BMEI0856). Furthermore, the entire NADH dehydrogenase operon was down-regulated in 16 MΔ*otpR*. The genes encoding the cytochrome D ubiquinol oxidase subunits I, II, and III (BMEII0759, BMEII0760, BMEI1899, BMEI1900, BMEI1901) were all down-regulated in 16 MΔ*otpR*. The NADH dehydrogenase operon and cytochrome D ubiquinol oxidase subunits participate in the oxidative phosphorylation, an important metabolic process for electron transport and energy release^45^.

We also found that the expression levels of five genes associated with nitrogen metabolism (*npd,* BMEII0460; *narI,* BMEII0953; BMEII0952; *nirV,* BMEII0987; *norF,* BMEII1000; and *norE,* BMEII1001) were altered in 16 MΔ*otpR* under acid stress. Meanwhile, two genes (BMEII0952, BMEII0953) participating in the denitrification pathway were up-regulated (Supplemental Data 2). *Brucella* applies denitrification metabolism to generate energy at a low-oxygen condition in an intracellular niche inside host macrophages^46^.

**Figure 3:**
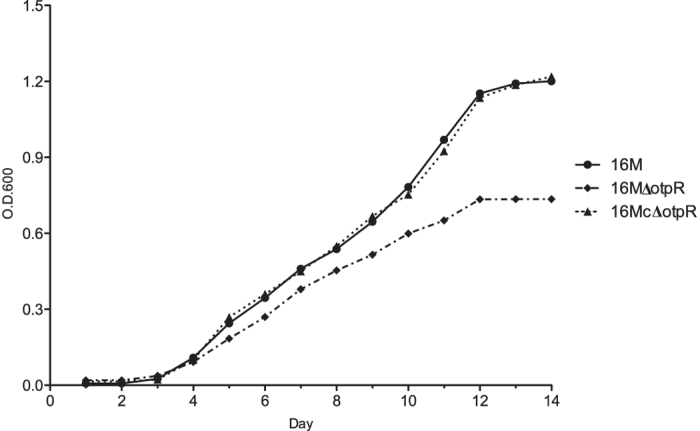
Growth curves of *the parental B. melitensis strain 16 M, its otpR mutant 16 MΔotpR, and the mutant complementation strain 16 McΔotpR* in a synthetic minimal medium. The minimal medium contains only carbon and nitrogen as the nutrient resources. Compared to the wild type strain, the *otpR* mutant had a decreased OD600 value. The gene complementation of the *otpR* mutant resumed the OD600 level. A curve of the optical density values at OD600 determined at several time points reflects the growth dynamics of a bacterial strain in the culture medium over the different time points.

To further investigate the importance of OtpR in regulating carbon and nitrogen metabolisms, we used a defined minimal medium that contains only carbon and nitrogen nutrients (without amino acids and growth factors). The minimal medium was used to separately culture parental strain 16 M, 16 MΔ*otpR,* and the mutant complementing strain 16 McΔ*otpR*, followed by the measuring of their dynamic growth profiles. All these three strains were able to grow in the minimal medium, indicating that the inorganic carbon and nitrogen resources provide sufficient nutrients for *Brucella* growth and replication. Compared to 16 M, the mutant 16MΔ*otpR* showed reduced growth at the late log phase (Fig. 3). The phenomenon suggested that OtpR was important to sustain regular cell growth through the regulation of the carbon and nitrogen metabolism. The observation was further confirmed by the complementation of the gene in the mutant as shown by the full recovery of the cell growth in 16 McΔ*otpR* (Fig. 3).

### OtpR regulates iron metabolism in *B. melitensis*

Our transcriptomics analysis also found that OtpR regulates many genes in iron metabolism (Supplemental Data 2). Compared to the 16 M, 16MΔ*otpR* mutant presented down-regulation of two ABC transporter systems. One ABC transporter system includes an ATP-binding protein DstD (BMEII0604, ATP/GTP-binding site-containing protein A) and a permease DstE (BMEII0606, ferric anguibactin transport system permease protein). This Dst protein–dependent ABC transporter is responsible for the utilization of iron by *B. melitensis* in low-iron medium^47^. Both *dstD* and *dstE* were also down-regulated in 16 MΔ*otpR.* The other ABC transporter system is the TonB-ExbB-ExbD complex that is critical to transport iron-siderophore complexes into bacterial cell. The TonB system is associated with 2, 3-dihydroxybenzoic acid assimilation in *B. melitensis* and allows adaptation to low-iron medium^47^. The expressions of both *exbB* (BMEI0365) and *exbD* (BMEI0366) were down-regulated in 16 MΔ*otpR*. Several other OtpR-regulated iron-related genes include *bfr* (BMEII0704, bacterioferritin), BMEII0584 (iron-binding periplasmic protein), BMEII0607 (ferric anguibactin-binding protein), *irrf2* (BMEII0707, RrF2 family protein), and *fecD* (BMEII0536, Fe^3+^dicitrate transport system permease protein fecd).

**Figure 4:**
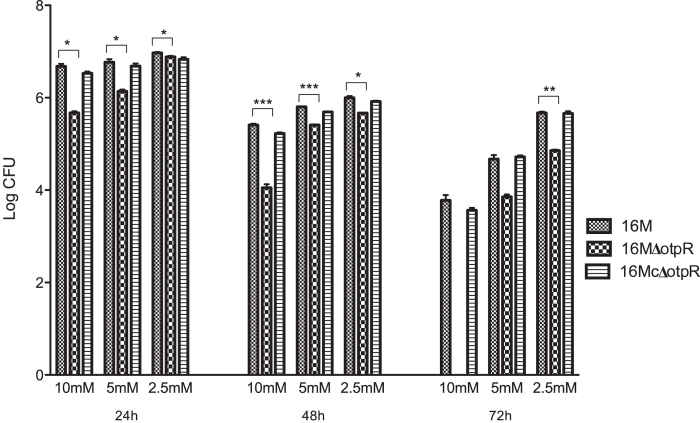
Survival of *B. melitensis* 16 MΔotpR, 16 McΔotpR, and the wild-type strain under iron-limited conditions. The survival of the strains was measured in the TSB medium containing 2.5 mM, 5.0 mM and 10.0 mM Fe^2+^ chelator 2,2`-dipyridyl. The measurements were taken at 24 h, 48 h and 72 h after inoculation. Experiments were performed in triplicates and a significant difference was observed. Statistical analysis was performed by comparing the CFUs of 16 M versus 16 M**Δ***otpR* bacteria at different time points using the Student’s *t*-test. *p* < 0.05 (*), *p* < 0.01 (**) and *p* < 0.001 (***) represents different levels of significant differences. It is noted that in the presence of 10.0 mM DIP, the *otpR* mutant could be detected at 24 and 48 h after inoculation, but not at 72 h. When the CFU/mL was determined, 200 μL of culture sample was used in each condition, which equals to a detection limit of 5 CFU/ml (or ~0.7 LOG CFU). It is possible that there were still some viable *otpR* mutant cells survived in the iron-limited medium at 72 h; however, the level of survival was below the laboratory’s detection limit.

To confirm that OtpR regulates iron metabolism, the tolerance of 16 MΔ*otpR* under an experimental condition of low iron was assessed after adding varying concentrations of the Fe^2+^ chelator DIP into the medium. In the presence of 2.5 mM, 5.0 mM, or 10 mM DIP, the survival capability of the mutant strain 16 MΔ*otpR* was less than its parental strain 16 M (Fig. 4), suggesting that OtpR is critical to the utilization of iron in the low iron medium. The *otpR* gene complementation of 16 MΔ*otpR* recovered the bacterial survival probably due to the recovered function of OtpR in the iron uptake. These results suggest that although the tolerance of 16 McΔ*otpR* to low-iron medium was similar to that of 16 M, the *otpR* mutant appeared to affect longer-term survival in iron-limited medium.

### OtpR regulates the expression of many known *Brucella* virulence factors and regulators

Many *Brucella* virulence-related genes were differentially expressed in 16 MΔ*otpR* under acid stress. All of the 12 Type IV secretion system genes in the *virB* operon were down-regulated in 16MΔ*otpR* (fold change > 4; Fig. 2 and Supplemental Data 2). This system is critical for the translocation of *Brucella* effectors to the host for trafficking into macrophages. As compared with the wild type, three genes associated with flagellar assembly, *flgG* (coding for flagellar basal-body components in the distal portion of the rod)*, flhA* (encoding a protein of the flagellar type III export apparatus), and *flgF* (coding for flagellar basal-body components), were down-regulated in the *otpR* mutant (Fig. 2).

Among the genes down-regulated in the *otpR* mutant under acid stress are 34 known transcriptional regulators including two quorum sensing regulators (Supplemental Data 2). The two quorum sensing regulators VjbR and BlxR may directly regulate specific biological processes in *Brucella*^48^,^49^,^50^. In addition, two other transcriptional regulators, PhoP and NorS, were up-regulated in the *otpR* mutant.

### RT-qPCR validates the RNA-Seq results of selected *B. melitensis* genes

To validate the data generated from the RNA-seq experiment, we repeated the acid induction experiment and used RT-qPCR assays to detect transcript levels of 22 genes that were down-regulated in 16 MΔ*otpR* and of 6 genes that were up-regulated in 16 MΔ*otpR*. Out of the 501 differentially expressed genes detested by our RNA-Seq analysis, these 28 genes were selected based on three criteria: (i) Gene function. We selected one or two genes that were differentially expressed from each functional group, *e.g.*, BMEI0655 belonging to ABC transporter system, BMEI1153 involved to oxidative phosphorylation, BMEI1325 belonging to a two-component system, and BMEII0704 associated with cell division. (ii) Virulence factor role. We purposely chose many important virulence-related genes out of the 501 gene list, such as BMEII0025 (*virb1*) and BMEII0035 (*virb11*) (T4SS components), and BMEII1116 (*vjbR*) (a quorum sensing-dependent transcriptional regulator). (iii) Gene position in the genome. These chosen 28 genes are located in different positions in the genome.

The mRNA levels of these 28 genes as determined by RT-qPCR were in good accordance with those from the RNA-Seq analysis (Table 1). Together, these results support the model that OtpR is critical in regulating *Brucella* virulence.

## Discussion

Our RNA-seq study found that under acidic stress, OtpR regulated 501 genes associated with many important functions, including metabolism, membrane transport, transcription, regulation, translation, and DNA replication and repair^51^,^52^,^53^,^54^. Many environmental stresses, such as heat and oxygen limitation, may affect the expression of genes associated with these functions^15^. However, the specific mechanisms of the gene regulations on these functions are unclear. Our results provide evidence to show that OtpR is an important *Brucella* regulator that regulates metabolism processes and bacterial virulence under acidic stress.

The identification of the critical role of OtpR in regulating a large number of genes involving metabolic processes expands our understanding of the gene in *Brucella* and possibly other bacteria (Fig. 2). Our previous study shows that *Brucella* OtpR regulates cell growth and cell morphology^8^. The OtpR homologue in *Caulobacter crescentus,* CenR, is also found to be important in regulating bacterial growth and cell cycle progression^55^. However, previous studies did not show the generic regulatory mechanism of OtpR in regulating the bacterial cell growth and cell cycle progression^8^. This study found some OtpR-regulated genes associated with cell cycle progression, and the maintenance of cell morphology. The iron acquisition within the host cell influences the capacity of *Brucella* to survive in a host^10^,^56^. This study first showed that OtpR regulated the iron metabolism. Compared to the parental strain and the complementation strain of 16 MΔ*otpR,* 16 MΔ*otpR* had a reduced ability to survive in low-iron media. It suggests that OtpR plays an important role in *B. melitensis* to survive in low-iron media under acidic stress or in normal conditions.

Importantly, this study demonstrated that OtpR regulated many genes related to *Brucella* virulence. The entire *virB* operon and 34 transcriptional regulators were significantly down-regulated in the *otpR* mutant, suggests that OtpR positively regulated the expression of these genes. The *virB* operon can be induced by acid stimuli or phagosome acidification^2^. Its expression is directly regulated by VjbR, BvrR, IHF and HutC through promoter binding^48^,^57^,^58^,^59^. This study showed that the transcriptional regulators VjbR and BlxR were also markedly down-regulated in the *otpR* mutant, especially VjbR. VjbR and BlxR in *Brucella* are the two quorum sensing-related LuxR-type factors that regulate the transcription of other genes, including the *virB* operon and genes for flagellar and outer membrane components^48^,^60^,^61^. Our findings suggested that OtpR might indirectly regulate VirB genes through direct interaction between OtpR and VjbR. Considering that *Brucella* survives in an acidic environment inside macrophages^10^, it was likely that once inside macrophages, OtpR becomes activated and regulate these virulence factors.

Considering OtpR is important in *Brucella* metabolism regulation and virulence, further research is required to analyze the OtpR-mediated regulatory mechanisms. Structural analysis revealed that OtpR contains a phosphoacceptor site, which suggests that it might belong to a two-component regulator system. Our amino acid sequence analysis found that OtpR is highly homologous to that of CenR in *Caulobacter crescentus.* In *C. Crescentus,* CenR is the regulator of a two-component system CenR/CenS that senses and acts on various environmental stimuli^55^. The CenS is the sensor of the CenR/CenS two-component system. Although a genome sequence analysis identified a possible gene homologous to CenS, our studies found that the gene does not act like a sensor for OtpR. More investigation is still required to identify the sensor of the OtpR regulator. Since many genes identified to be regulated by OtpR, it might be possible to use bioinformatics and experimental methods to predict and identify the binding site(s) of OtpR. Another area of research is to identify how OtpR interacts with and regulates this large number of *Brucella* genes. Instead of up- or down-regulating a large number of genes simultaneously, it is more likely that OtpR regulates these many genes through one or more defined pathways in a time-dependent matter. In addition to the acidic stress condition, other experimental factors may also regulate the functions of OtpR. The eventual discovery of the detailed OtpR-regulated interaction networks will be critical to understand *Brucella* pathogenesis and will support the rational design of therapeutic drugs and preventive vaccines.

In conclusion, through comparative transcriptome analysis, differential expressions of many genes, involving the carbohydrate metabolism, nitrogen metabolism, and iron metabolism, were observed in 16 MΔ*otpR* and its parental strain 16 M under acid stress. The results indicated that cell division proteins and iron metabolism could be regulated by OtpR, and several important virulence factors were also differentially expressed in 16 MΔ*otpR*. For examples, *virB* operon was significantly down-regulated, and the genes encoding for 36 known transcriptional regulators, including quorum-sensing regulators VjbR and BlxR were also down-regulated. Selective RNA-seq results were experimentally verified, which further deciphered the different phenomena associated with virulence, environmental stresses and cell morphology in 16 MΔ*otpR* and its parental strain 16 M. This study provided the important information for understanding the detailed OtpR-regulated interaction networks and *Brucella* pathogenesis.

## Additional Information

**How to cite this article**: Liu, W. *et al.* RNA-seq reveals the critical role of OtpR in regulating *Brucella melitensis* metabolism and virulence under acidic stress. *Sci. Rep.* **5**, 10864; doi: 10.1038/srep10864 (2015).

## Supplementary Material

Supplemental Data 1

Supplemental Data 2

## Figures and Tables

**Table 1:** 

***B. melitensis*****ORF**	**Gene name, predicted function**	**2**^**−ΔΔCt**^
BMEI0040	*gslc*,glutamate synthase [nadph] large chain	0.034
BMEI0655	ABC transporter ATP-binding protein	0.014
BMEI0747	*lsp*,lsu ribosomal protein l10p	0.022
BMEI1016	*aerobic*,fumarate hydratase class i	0.012
BMEI1153	NADH-quinone oxidoreductase chain f	0.019
BMEI1325	Sensory transduction protein kinase	0.039
BMEI1464	*pif*, protoheme ix farnesyltransferase	0.014
BMEI1652	Urease alpha subunit	0.022
BMEI1758	*blxR,* transcriptional activator	0.043
BMEI1855	aconitate hydratase	0.014
BMEII0025	*virb*1, type IV secretion system	0.011
BMEII0035	*virb*11, type IV secretion system	0.026
BMEII0071	*gntR*, transcriptional regulator	0.011
BMEII0245	*usp*, universal stress protein family	0.043
BMEII0409	*osmop,* Osmotically inducible protein c	0.016
BMEII0531	*fusB*,fusaric acid resistance protein	0.018
BMEII0704	Bacterioferritin	0.029
BMEII0742	*ftsK,* cell division protein	0.015
BMEII0953	Respiratory nitrate reductase 2 gamma chain	0.027
BMEII1047	*groES*,10 kDa chaperonin groES	0.013
BMEII1051	Fumarate hydratase c	0.046
BMEII1114	*flhB*, flagellar biosynthetic protein	0.013
BMEII1116	*vjbR*, transcriptional activator	0.023
BMEI1355	Hypothetical Cytosolic Protein	10
BMEI1751	Two component response regulator	4.62
BMEII0477	Uronate isomerase	3.58
BMEI1384	Transcriptional regulator, arac family	8.972
BMEI1766	Sulfite reductase (ferredoxin)	2.223

Validation of twenty-eight *otpR*-independent genes identified by RNA-Seq analysis. A 2^–∆∆Ct^ value >1 indicates that the gene was overexpressed in the *otpR* mutant, and a value of <1 indicates that the gene was expressed at a lower level in the mutant.
